# Pubertal timing and bone phenotype in early old age: findings from a British birth cohort study

**DOI:** 10.1093/ije/dyw131

**Published:** 2016-07-10

**Authors:** Diana Kuh, Stella G Muthuri, Adam Moore, Tim J Cole, Judith E Adams, Cyrus Cooper, Rebecca Hardy, Kate A Ward

**Affiliations:** 1 MRC Unit for Lifelong Health and Ageing at UCL, London, UK; 2 Population, Policy and Practice Programme, UCL Institute of Child Health, London, UK; 3 Clinical Radiology and Academic Health Science Centre, University of Manchester, Manchester, UK; 4 MRC Lifecourse Epidemiology Unit, University of Southampton Southampton, UK; 5 MRC Human Nutrition Research, Cambridge, UK

**Keywords:** Puberty, bone, birth cohort, life course

## Abstract

**Objectives**: To investigate the effect of pubertal timing, assessed in adolescence, on bone size, strength and density in men and women in early old age.

**Design**: A British birth cohort study with prospective indicators of pubertal timing based on age at menarche, clinical assessment of pubertal stage, and growth tempo from serial height measures, and bone measures derived from peripheral quantitative computed tomography (pQCT) and dual-energy X-ray absorptiometry (DXA) at 60-64 years of age among 866 women and 792 men.

**Methods**: A first set of regression models investigated the relationships between pubertal timing and bone size, strength and density, adjusting for current height and weight, smoking and adult socioeconomic position. To make an equivalent comparison between men and women, the percentage difference in bone outcomes was calculated for a 5-year difference in age at menarche, and in men a comparison between those who were fully mature or pre-adolescent at 14.5 years. A second set of models investigated the percentage difference in bone outcomes for a 5-year difference in timing of peak height velocity (height tempo) derived from longitudinal growth modelling (Superimposition by Translation and Rotation model; SITAR).

**Results**: After adjustment for current height and weight, a 5-year increase in age at menarche was associated with an 8% [95% confidence interval (CI) -17%, 0.5%, *P* = 0.07) lower trabecular volumetric bone mineral density (vBMD); men who were pre-adolescent at 14.5 years had a 9%, (95% CI -14%, -4%; *P* = 0.001) lower trabecular vBMD compared with those who had been fully mature. Other confounders did not attenuate these estimates further. Patterns of association were similar but somewhat weaker for lumbar spine and total hip areal BMD. Age at peak height velocity was associated with even larger differences in BMD in men and women, and was negatively associated with bone size and strength.

**Conclusions**: The association between later puberty and lower BMD persists into early old age. The 9-10% lower trabecular vBMD in later compared with earlier maturers could be clinically important given a rate of bone loss from midlife of 1-2% a year and the negative association between BMD and fracture.

## Introduction

Puberty is an important period for longitudinal and appositional bone growth and mineral accrual: 20-30% of an individual’s total body bone mineral is accrued during the pubertal growth spurt.^1^^,^^2^ It follows that ensuring optimal growth during this period will be important for future bone health and fracture risk.^3^^,^^4^ The extent to which pubertal timing is related to later bone phenotype and fracture risk has been investigated in a number of studies of different types.^5^ Most recently, a large genomic analysis revealed genetic correlations between timing of puberty in men and women and a range of health outcomes, including an inverse correlation with areal bone mineral density (aBMD) of the lumbar spine in 33 000 individuals.^6^ Such studies have the added benefit of avoiding any problems of bias due to retrospective recall of pubertal timing.^7^ This study supports the findings of some (for example ^8–10^), but not all^11^ retrospective epidemiological studies of premenopausal and postmenopausal women that have shown that later age at menarche is associated with reduced aBMD and increased fracture risk. The most recent of the retrospective epidemiological studies is based on over 250 000 women from UK Biobank and reported a reduced risk of self-reported doctor-diagnosed osteoporosis for those with an early menarche;^12^ however, in men from the same study, recalled timing of voice breaking was not strongly associated with osteoporosis risk.^12^

Prospective longitudinal studies with gold-standard bone phenotyping are required to fully understand relationships between pubertal timing, the development of peak bone mass, and bone architecture and fracture risk. There are a number of highly informative longitudinal studies,^2^^,^^13–17^.but none have follow-up beyond the third decade of life. Findings from studies using peripheral quantitative computed tomography (pQCT) or high-resolution pQCT have reported small but significant, negative associations between cortical thickness, medullary area and bone failure load in young women and age at menarche.^13^^,^^14^ Pubertal timing in younger cohorts, and especially in men, has more often been assessed using measures of peak height velocity (PHV), as clinical assessment of the Tanner stages of physical development is rare.^2^^,^^15^^,^^16^^,^^18^ Later age at PHV was associated with lower trabecular and cortical volumetric BMD (vBMD) and with total body and radius aBMD, in Swedish men aged 19 years;^16^^,^^18^ but by 24 years, there had been substantial catch-up and only deficits in aBMD and vBMD of the radius remained. These findings suggest that any differences in bone due to timing of puberty may be attenuated once catch-up growth has occurred.^17^

The MRC National Survey of Health and Development (NSHD), the oldest British birth cohort, initiated in 1946, has markers of pubertal maturation based on clinical assessments in adolescence, serial growth measures, and bone measures at 60-64 years derived from pQCT, as well as dual-energy X-ray absorptiometry (DXA), in a large sample of men and women. We have previously described in this cohort associations between height and weight gain at different stages of growth, and bone phenotype at 60-64 years.^19^ Some of those differences described may have been due to pubertal timing. Therefore the first aim of the current study was to investigate the effect of age at menarche and pubertal stage, acquired during adolescence, on pQCT and DXA-derived bone outcomes in early old age. The second aim was to compare the these relationships with those between pubertal growth markers of tempo, derived using an instrument for longitudinal growth curve analysis called the Superimposition by Translation and Rotation (SITAR) model,^20^^,^^21^ and the bone parameters.

## Methods

### Sample

The NSHD is cohort study of 2815 men and 2547 women followed up since their birth in a week in March 1946 in England, Scotland and Wales. At the 24th follow-up, when study members were aged between 60 and 64 years, 2856 were still alive and had a known current address in mainland Britain. Participants were invited for assessment at one of six clinical research facilities (CRFs); those unable or unwilling to travel were offered a home visit by a research nurse.^22^ A total 2229 participants out of the 2856 invited (78%) underwent assessment: 1690 attended a CRF and the remaining 539 were seen in their homes.^23^ A total of 778 participants had died. Of the remaining participants, 570 were living abroad, 594 had previously withdrawn from the study and 564 were lost to follow-up.

### Bone health assessment at 60-64 years

Of those attending a CRF, 792 men and 866 women underwent a DXA and 658 men and 697 women had a pQCT scan of the radius (non-dominant side). DXA scans were acquired in all six CRFs using the QDR 4500 Discovery (Hologic Inc, Bedford, MA), and in five CRFs pQCT data using a XCT 2000 (Stratec, Pforzheim, Germany) scanner were additionally collected. Details of scan acquisition and cross-calibration have been previously described.^19^ Standard manufacturer protocols were followed for data acquisition. Machine variability between centres was monitored using the European Spine Phantom and the pQCT scanners using the European Forearm phantom and, where necessary, cross-calibration was performed. Standard manufacturer procedures were followed for daily Quality Assurance/Quality Control and all phantom and scan analyses were centralized to one centre (JEA) for grading, analysis and collation of a harmonized database. Repeat precision was determined in one centre and was < 1% for DXA measurements, and for pQCT ranged between 1% and 3%.

The bone outcomes for this analysis were pQCT-derived measures at the radius distal 4% site of trabecular and total vBMD and distal cross-sectional area (CSA), and at the radius 50% site of CSA of the diaphysis and the medullary cavity (medullary CSA), cortical vBMD and polar strength strain index (SSI), an *in vivo* estimate of bone strength.^24^ DXA-derived measurements of areal BMD for lumbar spine (L1 L4) and total hip were also obtained.

### Pubertal timing

Reports of pubertal timing were obtained in 1961 when study members were aged 14-15 years (mean 14.5, range 14.3-15.2 years), when they underwent a medical examination and interview by a school doctor.^25^ Age at menarche was obtained from mothers’ reports at the examination. For 94 of the 188 girls who had not reached menarche by the time of the examination, retrospective reports from woman study members were later obtained from a postal questionnaire at age 48. Age at menarche in years was used for descriptive analyses, and modelled as months since birth. In boys, the school doctor assessed: the development of genitalia (advanced or complete, early or infantile); voice breaking (completely broken, starting to break, not yet started); visible pubic hair (profuse, sparse, none); and visible axillary hair (yes or no). Based on these observations, boys were classified as fully mature (advanced development of genitalia, profuse pubic hair and axillary hair, and voice broken), advanced puberty (advanced development of genitalia, but at least one other indicator not fully mature), early puberty (early development of genitalia, and some pubic or axillary hair or voice starting to break) and pre-adolescent (infantile genitalia or early adolescent genitalia, no pubic or axillary hair and voice not broken).^26^

Individual patterns of height growth during puberty were estimated using the SITAR model of growth curve analysis.^20^^,^^21^ Data were collected using standardized protocols at ages 2, 4, 6, 7, 11 and 15, and self-reported at ages 20 and 26. To provide additional information at intermediate ages, the NSHD data were augmented by height data between 5 and 19 years from the ALSPAC cohort,^27^ as described by Cole *et al.*^21^ The SITAR model summarizes each individual’s growth curve in terms of three parameters: size, tempo and velocity, each expressed relative to the mean curve. The model is estimated as a mixed effects growth model with a cubic B-spline mean curve, including both fixed and random (subject-specific) effects for size, tempo and velocity. For the purposes of this paper, we present only height tempo data, as these indicate the timing of puberty which is the focus of the current paper. A negative height tempo indicates earlier puberty, positive indicates later puberty. Details of the other SITAR variables are given in Cole *et al.*^21^

### Covariables

Current body size was assessed by height (m) and weight (kg), according to a standard protocol. Smoking was split into two categories, cigarette smokers versus non-smokers at 60-64 years. According to the Registrar General’s social class classification, social class was categorized based on the participant’s occupation at age 53 years (or at other ages if missing, *n* = 4) to split those who were in the manual social classes from those in the non-manual social classes in adulthood. Other potential confounders or mediators that were investigated in additional analyses were: leisure-time physical activity (distinguishing those who were most active (reporting vigorous leisure time activity more than fibe times a month) from those less active (one to four times a month) or inactive;^28^ and certain health conditions assessed at age 60-64 years and detailed elsewhere.^29^ We included a set of cardiometabolic health conditions (cardiovascular disease, hypertension, raised cholesterol and diabetes) and a second set of conditions (liver disease, thyroid disease and psychiatric problems) that may be relevant for bone.

In women, age when periods ceased naturally or because of hysterectomy or bilateral oophorectomy was obtained from information on menstrual irregularity and date of last menstrual period or any operation to remove the uterus or ovaries. Information was collected in annual postal questionnaires from age 47 to 54 years and at 57 years, and from face-to-face interviews with trained research nurses at 43, 53 and 60-64 years of age.^30^

### Statistical analysis

Of those who had a DXA or pQCT scan, 75% also had reports of pubertal timing. R version 3.2 (www.R-project.org) was used to fit the SITAR model and generate the SITAR random effects. For all other analyses, Stata v10.1 was used. Regression models used natural logarithms for all bone variables for comparative purposes. The coefficients from these models are presented as the percentage difference in the bone outcome by category, or per unit increase.

### Age at menarche and pubertal stage

Initial adjustments were for current body size (height and weight at 60-64 years), and then for current smoking and adult social class. In women, additional adjustments were made for age at period cessation in a subset of women where those data were available. In a set of further analyses, we additionally adjusted for physical activity, cardiometabolic conditions, and liver disease, thyroid disease and psychiatric problems. We also re-ran the analyses excluding 11% with osteoporosis, based on bone density T-score from the DXA scan ≤ 2.5 at spine, femoral neck or hip.^29^

To make an equivalent comparison between men and women that compared those with the earliest and latest pubertal timing, we calculated the percentage difference in the bone outcomes for a 5-year difference in age at menarche and the percentage difference between men who were fully mature and pre-adolescent at age 14.5 years.

### SITAR analysis of height tempo

Using the same participants with reported pubertal timing, the percentage difference in each of the bone outcomes by the derived SITAR parameter of height tempo was derived. The models first included height tempo unadjusted, and then additionally included current height and weight. To compare the estimates for tempo with those based on reported pubertal timing, we calculated the percentage difference for a 5-year difference in timing of puberty for women (10.5 to 15.5 years) and men (11.5 to 16.5 years).

## Results

In the sample of 704 women and 655 men with at least one bone outcome and reported pubertal timing, mean age at menarche was 13.0 years (standard deviation (SD) 1 year, 2 months); and by 14.5 years, 26% of the boys were fully mature, 30% were advanced, 34% were at an early stage and 10% were still pre-adolescent. Descriptions of the bone outcomes, puberty indicators and covariables are shown in Table 1.

**Table 1.** Characteristics of 655 men and 704 women from the MRC National Survey of Health and Development with at least one bone measure and information on age at menarche or pubertal stage

Measures	Men		Women	
	No.	Mean (SD)	No.	Mean (SD)
pQCT measures				
pQCT-cortical sites				
50% radius				
Diaphysis CSA (mm^2^)	547	155 (23)	571	113 (16)
Medullary CSA (mm^2^)	546	43 (14)	569	35 (12)
Polar SSI (mm^3^)	543	348 (70)	572	211 (43)
pQCT-trabecular sites				
Distal radius (4%)				
Distal CSA (mm^2^)	547	171 (34)	566	133 (24)
pQCT-50% radius				
Cortical vBMD (mg/cm^3^)	547	1159 (35)	572	1148.2 (39)
pQCT-distal radius (4%)				
Trabecular vBMD (mg/cm^3^)	546	205 (41)	565	173 (42)
Total density vBMD (mg/cm^3^)	547	391 (67)	566	332 (71)
DXA measures				
DXA aBMD (g/cm^2^)				
Spine L1-L4 aBMD	652	1.05 (0.18)	699	0.95 (0.16)
Total hip aBMD	645	1.00 (0.14)	695	0.87 (0.13)
Current body size				
Height (m) at 60-64 years	655	1.75 (0.06)	704	1.62 (0.06)
Weight (kg) at 60-64 years	655	85 (13.1)	704	72 (14.2)
Pubertal timing indicators				
Age at menarche (years months)	n/a		704	13years 0 months (1year 2 months)
		**%**	**%**	**%**
Age at menarche	n/a			
9-10			24	3.4
11			91	12.9
12			214	30.4
13			243	34.5
14			98	13.9
15-19			34	4.8
Total (= 100%)			704	
Pubertal stage (14.5 year)				
Fully mature	168	25.6		
Advanced	200	30.5		
Early	222	33.9		
Pre-adolescent	65	9.9		
Total (= 100%)	655			
Development of genitalia				
Advanced or complete	368	56.2		
Early	263	40.1		
Pre-adolescent	24	3.7		
Total (= 100%)	655			
Broken voice				
Completely broken	245	37.6		
Starting to break	235	36.0		
Not yet broken	172	26.4		
Total (=100%)	652			
Pubic hair				
Profuse	313	47.9		
Sparse	263	40.3		
None	77	11.8		
Total (= 100%)	653			
Axillary hair				
Yes	368	56.5		
No	283	43.5		
Total (= 100%)	651			
Covariables				
Smoking				
No	584	89.9	626	89.4
Yes	66	10.1	74	10.6
Total (=100%)	650		700	
Own adult social class				
Non-manual	473	72.2	562	79.8
Manual	182	27.8	142	20.2
Total (= 100%)	655		704	
Age at period cessation (years)				
27-39			40	7.0
40-44			57	9.9
45-49			131	22.9
50-52			159	27.7
53-55			126	22.0
56-62			60	10.5
Total (= 100%)			573	

### Mean differences in bone size, density and strength by pubertal timing

In women, age at menarche was not associated with any measures of CSA (diaphysis, medullary or distal radius); nor was it associated with polar SSI (Table 2a). However, later age at menarche was associated with lower total and trabecular vBMD and lumbar spine and total hip aBMD (Table 2a).

**Table 2a.** Mean and standard deviation (SD) for pQCT-derived outcomes at 60-64 years by age at menarche, women

		Age at menarche							
		9-10	11	12	13	14	15-19	Totalsample	***P*-value** *
	No.	Mean (SD)	Mean (SD)	Mean (SD)	Mean (SD)	Mean (SD)	Mean (SD)	Mean (SD)	
pQCT									
Distal CSA mm^2^ (4%)	566	132 (23)	132 (24)	130 (24)	134 (25)	136 (24)	129 (24)	133 (24)	0.3
Diaphysis CSA mm^2^ (50%)	571	111 (12)	113 (17)	115 (17)	111 (16)	112 (14)	112 (18)	113 (16)	0.3
Medullary CSA mm^2^ (50%)	570	31 (9)	34 (13)	37 (13)	36 (12)	35 (12)	35 (13)	35 (12)	0.7
Polar SSI mm^3^ (50%)	572	212 (34)	217 (45)	214 (45)	207 (42)	208 (39)	211 (39)	211 (43)	0.1
Total vBMD mg/cm^3^ (4%)	566	351 (82)	339 (69)	340 (74)	322 (70)	318 (64)	337 (69)	332 (71)	0.01
Trabecular vBMD mg/cm^3^ (4%)	565	178 (40)	177 (41)	179 (46)	169 (41)	168 (35)	158 (40)	173 (42)	0.004
Cortical vBMD mg/cm^3^ (50%)	572	1157 (33)	1146 (44)	1146 (40)	1149 (40)	1152 (33)	1151 (40)	1148 (39)	0.5
DXA									
Lumbar spine aBMD g/cm^2^	699	0.98 (0.14)	0.98 (0.18)	0.95 (0.16)	0.95 (0.16)	0.92 (0.15)	0.91 (0.16)	0.95 (0.16)	0.006
Total hip aBMD g/cm^2^	695	0.90 (0.14)	0.90 (0.13)	0.87 (0.12)	0.86 (0.13)	0.85 (0.12)	0.84 (0.12)	0.87 (0.13)	0.001

*P-values from regression models with logged bone outcomes and including age at menarche in months since birth as a continuous variable.

In men, early puberty at 14.5 years was associated with smaller diaphysis and medullary CSA, but not distal CSA, and with lower polar SSI (Table 2b). Later pubertal maturation was associated with lower mean values of trabecular vBMD and lumbar spine and total hip aBMD, and higher mean values of cortical vBMD; but there were no differences in total vBMD. All the indicators of pubertal maturation (axillary hair, pubic hair and voice breaking) showed similar patterns with these bone parameters (Supplementary Data, available as Supplementary Data at *IJE* online).

**Table 2b.** Mean and standard deviation (SD) for pQCT-derived and DXA-derived outcomes at 60-64 years by pubertal stage at 14.5 years, men

		Pubertal stage					
		Fully mature	Advanced	Early	Pre-adolescent	Total sample	*P*-value
	No.	Mean (SD)	Mean (SD)	Mean (SD)	Mean (SD)	Mean (SD)	
pQCT							
Distal CSA mm^2^ (4%)	547	172 (38)	174 (31)	170 (33)	167 (32)	171 (34)	0.3
Diaphysis CSA mm^2^ (50%)	547	159 (25)	155 (21)	152 (22)	153 (22)	154 (23)	0.01
Medullary CSA mm^2^ (50%)	546	45 (16)	44 (14)	41 (12)	41 (14)	43 (14)	0.02
Polar SSI mm^3^ (50%)	543	358 (77)	349 (64)	342 (72)	340 (61)	348 (70)	0.03
Total vBMD mg/cm^3^ (4%)	547	403 (71)	384 (67)	388 (64)	397 (71)	391 (67)	0.3
Trabecular vBMD mg/cm^3^ (4%)	546	216 (43)	205 (41)	201 (40)	197 (41)	205 (41)	< 0.001
Cortical vBMD mg/cm^3^ (50%)	547	1153 (37)	1159 (35)	1161 (33)	1162 (31)	1159 (35)	0.03
DXA							
Lumbar spine aBMD g/cm^2^	652	1.08 (0.18)	1.04 (0.19)	1.05 (0.17)	1.01 (0.15)	1.05 (0.18)	0.02
Total hip aBMD g/cm^2^	645	1.02 (0.14)	1.00 (0.16)	0.99 (0.14)	0.97 (0.15)	1.00 (0.14)	0.01

*P-values from regression models with logged bone outcomes and including pubertal stage as a continuous variable.

### Regression models for age at menarche and pubertal stage

In women with complete data (*n* = 573 for pQCT-derived measures and 704 for DXA-derived measures), a 5-year increase in age at menarche was associated with an 11% (95% CI -19%, -3%, *P* = 0.01) lower trabecular vBMD (Table 3a, model 1). Adjusting for current height and weight attenuated this estimate somewhat (-8%, 95% CI -17%, 0.5%; *P* = 0.07) (Table 3a, model 2). There was no further attenuation after adjusting for smoking and social class (Table 3a, model 3). The associations for total vBMD and aBMD in lumbar spine and total hip were in the same direction but slightly weaker; no other associations were observed. In the subset of women with age at period cessation, the association between age at menarche and BMD was attenuated after adjustment for body size, particularly for trabecular vBMD, but not on further adjustment for age at period cessation or other confounders (Supplementary Data, available as Supplementary Data at *IJE* online).

**Table 3a.** Percentage difference in DXA-derived and pQCT-derived outcomes for a 5-year difference (diff) in age at menarche , additionally adjusted for current height and weight, smoking and adult social class, women

		Unadjusted			Adjusted for height & weight			Additionally adjusted for smoking and adult social class		
	No.	% diff	95% CI	*P*-value	% diff	95% CI	*P*-value	% diff	95% CI	*P*-value
pQCT measures										
pQCT-cortical sites										
50% radius										
Diaphysis CSA (mm^2^)	567	−2.5	−7.1, 2.12	0.3	−3.3	−7.5, 1.0	0.1	−3.3	−7.6, 0.9	0.1
Medullary CSA (mm^2^)	565	0.8	−10.4, 12	0.9	−2.2	−13.4, 8.9	0.7	−2.5	−13.7, 8.7	0.7
Polar stress strain index (mm^3^)	568	−4.1	−10.6, 2.5	0.2	−4.9	−11.1, 1.2	0.1	−4.9	−11.1 to1.3	0.1
pQCT-trabecular sites										
Distal radius (4%)										
Distal CSA (mm^2^)	562	3.6	−2.7, 9.9	0.3	2.0	−4.1, 8.0	0.5	2.1	−3.9, 8.2	0.5
pQCT-50% radius										
Cortical vBMD (mg/cm^3^)	568	0.2	−0.9, 1.4	0.7	0.4	−0.8, 1.5	0.5	0.4	−0.7, 1.6	0.5
pQCT-distal radius (4%)										
Trabecular vBMD (mg/cm^3^)	561	−11	−19.3, -2.5	0.01	−8.0	−16.6, 0.5	0.07	−8.1	−16.7, 0.5	0.07
Total density vBMD (mg/cm^3^)	562	−8	−15.1, -0.9	0.03	−5.5	−12.7, 1.7	0.1	−5.7	−12.9, 1.5	0.1
DXA measures										
DXA aBMD (g/cm^2^)										
Spine L1-L4 aBMD	695	−7.2	−12.4, -2.1	0.006	−4.8	−9.9, 0.3	0.06	−4.8	−9.9, 0.3	0.06
Total hip aBMD	691	−8.2	−12.6, -3.8	< 0.001	−4.3	−8.3, −0.3	0.04	−4.2	−8.1 to−0.1	0.04

In men (*n* = 550 for pQCT-derived measures and 655 for the DXA-derived measures), the differences in CSA between those assessed as pre-adolescent compared with those fully mature was greatest for medullary CSA (-10%, 95% CI -19%, -1%; *P* = 0.02); adjusting for height and weight attenuated these differences, but additionally adjusting for smoking and social class did not (Table 3b, models 1-3). The corresponding difference in trabecular vBMD was -9% (95% CI -15%, -4%; *P* = 0.001), with almost no attenuation after adjustment for current height and weight (-9%, 95% CI -14%, -4%; *P* = 0.001) or for smoking and social class (Table 3b models 1-3). In contrast, those assessed as pre-adolescent had higher cortical vBMD (0.9%, 95% CI 0.1, 1.7, p = 0.02); this was not attenuated after further adjustment. Negative associations were also seen for aBMD in lumbar spine and total hip, but these were attenuated after adjustment for confounders. The difference in polar SSI was -6% (95% CI -11%, -0.8%; *P* = 0.02), and this was also reduced after adjustment for body size (-4%, 95% CI -9%, 0.5%; *P* = 0.08) but not after adjusting for smoking and social class (Table 3b, models 1-3). Regression models substituting pubertal stage for the separate pubertal indicators in turn (axillary hair, public hair, genitalia and broken voice) all showed similar patterns. The results remained the same upon additional adjustments for physical activity and certain health conditions (cardiometabolic disorders, liver disease, thyroid disease or psychiatric problems). Excluding those with osteoporosis had only a minor effect on the estimates.

**Table 3b.** Percentage difference in DXA-derived and pQCT-derived outcomes comparing pre-adolescent with fully mature males at 14.5 years, additionally adjusted for current height and weight, smoking and adult social class, men

		Unadjusted			Adjusted for height & weight			Additionally adjusted for smoking and adult social class		
	No.	% diff	95% CI	*P*-value	% diff	95% CI	*P*-value	% diff	95% CI	*P*-value
pQCT measures										
pQCT-cortical sites										
50% radius										
Diaphysis CSA (mm^2^)	542	−5.0	−8.7, -1.2	0.009	−3.8	−7.1, −0.4	0.03	−3.8	−7.1, −0.5	0.03
Medullary CSA (mm^2^)	541	−9.9	−18.6, -1.3	0.02	−8.4	−16.8, −0.02	0.05	−8.5	.03	0.046
Polar stress strain index (mm^3^)	538	−6.1	−11.4, -0.8	0.02	−4.3	−9.0, 0.5	0.08	−4.4	−9.0, 0.3	0.07
pQCT-trabecular sites										
Distal radius (4%)										
Distal CSA (mm^2^)	542	−2.7	−8.1, 2.7	0.3	−1.8	−7.1, 3.5	0.5	−1.7	−6.9, 3.6	0.5
pQCT-50% radius										
Cortical vBMD (mg/cm^3^)	542	0.9	0.1, 1.7	0.02	0.8	0.1, 1.6	0.04	0.8	0.02, 1.6	0.04
pQCT-distal radius (4%)										
Trabecular vBMD (mg/cm^3^)	541	−9.2	−14.7, -3.8	0.001	−8.9	−14.4, −3.5	0.001	−9.2	−14.6 to−3.7	0.001
Total density vBMD (mg/cm^3^)	542	−1.5	−6.0, 3.1	0.5	−1.3	−5.9, 3.2	0.6	−1.6	−6.1, 3.0	0.5
DXA measures										
DXA aBMD (g/cm^2^)										
Spine L1-L4 aBMD	647	−4.5	−8.5, -0.4	0.03	−2.7	−6.4, 1.1	0.2	−2.6	−6.4, 1.2	0.2
Total hip aBMD	640	−4.5	−8.0, -1.0	0.01	−2.5	−5.6, 0.7	0.1	−2.6	−5.7, 0.5	0.1

**Table 4.** Percentage difference (diff) in DXA-derived and pQCT-derived outcomes for a 5-year difference in height tempo

	Women						Men					
	Height tempo						Height tempo					
	Unadjusted			Adjusted for height & weight			Unadjusted			Adjusted for height & weight		
	% diff	95%CI	*P*-value	% diff	95%CI	*P*-value	% diff	95% CI	*P*-value	%diff	95% CI	*P*-value
pQCT measures												
pQCT-cortical sites												
50% radius												
Diaphysis CSA (mm^2^)	−4.9	−10.6, 0.7	0.09	−6.9	−12, −1.8	0.009	−6.9	−13.5, -0.3	0.04	−8.8	−14.8, -2.8	0.004
Medullary CSA (mm^2^)	−1.7	−15.9, 12.5	0.8	−6.4	−20.5, 7.7	0.4	−16.5	−31.8, -1.2	0.04	−19.3	−34.2, -4.4	0.01
Polar stress strain index (mm^3^)	−7.5	−15.6, 0.7	0.07	−9.8	−17.3, -2.3	0.01	−8.3	−17.5, 1	0.08	−10.9	−19.3, -2.4	0.01
pQCT-trabecular sites												
Distal radius (4%)												
Distal CSA (mm^2^)	1.6	−6, 9.2	0.7	−1.7	−8.9, 5.5	0.7	3.2	−6.5, 12.9	0.5	1.9	−7.6, 11.4	0.7
pQCT-50% radius												
Cortical vBMD (mg/cm^3^)	−0.6	−2, 0.8	0.4	−0.5	−1.9, 0.9	00.5	0.7	−0.7, 2.1	0.3	0.6	−0.8, 2	0.4
pQCT-distal radius (4%)												
Trabecular vBMD (mg/cm^3^)	−22.2	−32.7, −11.8	< 0.001	−18.4	−28.9, −7.9	0.001	−21.4	−31.2, 11.6	< 0.001	−20.6	−30.4, −10.8	< 0.001
Total density vBMD (mg/cm^3^)	−11.0	−19.7, −2.4	0.01	−7.5	−16.2, 1.1	0.09	−9.6	−17.5, -1.7	0.02	−9.2	−17.1, -1.2	0.02
DXA measures												
DXA aBMD (g/cm^2^)												
Spine L1-L4 aBMD	−13.9	−20.4, −7.5	< 0.001	−11.8	−18, −5.5	< 0.001	−9.7	−16.9, −2.4	0.009	−9.7	−16.6, −2.9	0.006
Total hip aBMD	−9.9	−15.5, −4.2	0.001	−5.9	−10.9, −0.9	00.02	−7.0	−13.2, −0.9	.03	−6.4	−11.9, −0.8	0.02

### Regression models for SITAR growth parameters

**Figure 1:**
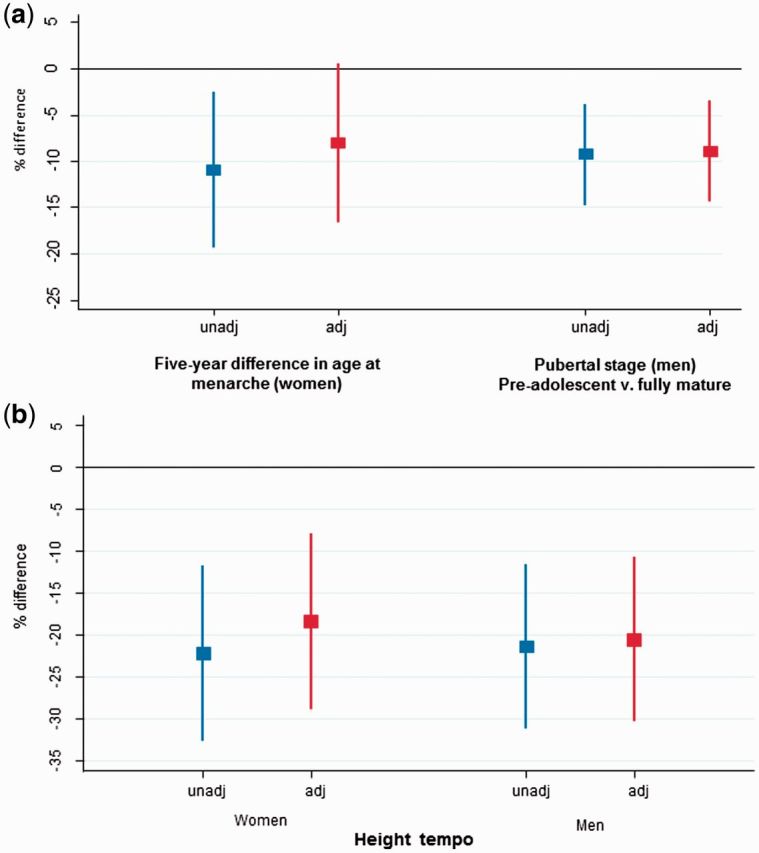
Percentage difference in peripheral trabecular vBMD by a) pubertal timing and b) height tempo for women and men unadjusted and adjusted for height and weight.

The negative coefficients for height tempo in men and women are consistent with our findings of later pubertal timing, being associated with lower trabecular vBMD, and spine and total hip aBMD (Table 4). Height tempo was also associated with total and trabecular vBMD, and with spine and total hip aBMD in men. In most cases the height tempo effects were larger than those based on reported pubertal timing, though the confidence intervals were also wider and the significance levels broadly similar (Figure 1). Height tempo in men and women was inversely associated with diaphysis CSA and polar SSI; additionally, in men it was negatively associated with medullary CSA. These associations were attenuated by adjustment for current body size in men but not women.

## Discussion

In a large British birth cohort, later puberty, determined at clinical assessments in adolescence, was associated with lower trabecular vBMD at the radius and lower aBMD at the lumbar spine and hip in early old age. The effect was seen across the range of pubertal maturation and was of a similar size in men and women, explaining 9-10% of the variation in vBMD between the earliest and latest maturers. In men but not women, later puberty was also associated with reduced bone size and strength, although these associations were weaker than for vBMD. Replacing these pubertal indicators with SITAR-derived height tempo gave similar results and the associations were, on the whole, stronger. Later puberty was again negatively associated with trabecular vBMD and aBMD at both sites. In contrast to the analysis with reported age at menarche, bone area and strength were also lower in women with later pubertal growth. Similar relationships for bone size and strength were found in men.

Of the variance in BMD, 60-80% is explained by genetics,^5^^,^^6^^,^^31^^,^^32^ which could explain the close relationships between vBMD and tempo or pubertal timing. During growth, the cross-section of a bone will also be determined by allometric scaling to height growth and to weight gain.^21^ Using SITAR in both men and women, later puberty was associated at the diaphysis, with a smaller, less strong, bone, presumably because of the extended limb-growth period in these individuals. Medullary CSA was also smaller in later-maturing men, indicating proportional growth of the periosteal and endosteal surfaces. In women, no associations with medullary CSA existed. There are two possible explanations for this. First, the lack of expansion of the medullary cavity in females during pubertal growth is often attributed as preparation for pregnancy, being less driven by biomechanical adaptation to height growth as is the case in men. Second, at the time of measurement this group of women were all post-menopausal. Bone loss with ageing occurs at the endosteal surface, and so any relationships between puberty and medullary area may have been attenuated by bone loss that had already occurred.

These are important findings as, once skeletal maturity is reached and longitudinal growth ceases, bones grow up to 2 years more in width and 4 years more in mineral content,^2^and thereafter the skeletal reservoir is set for adult life. As such, the amount of bone an individual has at the end of growth is a determinant of future fracture risk.^3^^,^^4^ Further to this, the vBMD-tempo association is interesting in that vBMD was ∼ 20% less in those with late compared with early puberty. Given that a 1-SD reduction in BMD results in a doubling of fracture risk,^33^ the differences in the current study may represent a significant increased risk of fracture between the two extremes. There are fewer data relating SSI to fracture risk, though in men with fractures compared with those without, SSI was associated with incident fracture (hazard ratio 2.3, 95% CI 1.3, 4.1).^34^

The main difference between the two methods of assessment for pubertal timing is that we used a single event to mark puberty, whereas the SITAR analysis used longitudinal growth data. This difference in characterization of pubertal growth may explain why more associations were found between height tempo and bone size and strength than for measures of reported pubertal timing. Because the tempo parameter modelled pubertal timing based on the whole of adolescent growth, and because longitudinal growth is a more proximal indicator of growth in bone cross-section and mineral apposition than the development of secondary sexual characteristics, these differences may have a biological basis and reflect the differential impact of sex hormones and other endocrine factors, rather than simply being due to errors of recall or measurement. Being able to compare these two methods of assessment and relationships with bone is a unique feature of the NSHD. A second advantage of this modelling technique is that men and women were aligned on the same developmental scale to determine timing, which may have improved accuracy of relationships described.

As in the current study, later menarche is associated with lower aBMD in some studies.^8^^,^^9^^,^^13^^,^^14^ Since then there have been some prospective studies that have used pQCT and followed women into late adolescence or early-mid adulthood.^35^ Again, results were not conclusive. The most consistent finding was that later age of menarche was associated with greater endosteal circumference (i.e. medullary area) and thinner cortices. A study using high-resolution pQCT reported a negative association between total and cortical vBMD. This is in contrast to our findings which found no association in older women between timing of puberty and cortical bone outcomes.

The UK Biobank study is the largest to date to report the relationship between pubertal timing and osteoporosis risk. In women, self-reported puberty was categorized into early, normal and late. In the late puberty group, incidence of self-reported osteoporosis was increased, and in the early group it was reduced. This confirms previous epidemiological studies showing late menarche to be a risk factor for fracture.^36^ The associations found in our study would be consistent with these findings of increased risk of fractures and osteoporosis.

In males in the GOOD study, with the exception of cortical vBMD, later age at PHV was not associated with any DXA or pQCT outcome at age 23-25 years (around the age of peak bone mass).^17^ This is in contrast to the current study, which showed associations particularly between pubertal timing and bone size and distribution at the cortical site. In the UK Biobank, no associations were found between reported time of voice breaking and self-reported osteoporosis.^12^

## Strengths and limitations

The main strength of this study is that it is the only one to have collected growth and pubertal information prospectively and to have been able to relate those measures to gold-standard bone outcomes in early old age. Second, pQCT as well as DXA measurements were taken and results were consistent at both weight-bearing sites (lumbar spine and total hip by DXA) and non weight-bearing sites (radius by pQCT). Peripheral QCT has advantages over DXA in that measures are volumetric and not size dependent, and separate measures of trabecular and cortical compartments allow assessment of the size and distribution of bone, which are important predictors of bone strength. Third, this relatively large British sample included men and women, and the narrow age range of the sample at assessment limits potential confounding by age.

One limitation is that bone was only measured in early old age, so we do not know whether the associations with various bone outcomes were the result of peak bone mass or adult bone loss, although we (and others^5^) would hypothesize that the former would be more likely. A second limitation is that we cannot translate our findings of pubertal timing and future bone phenotype to fracture risk until sufficient events have accrued; however, it is known that lower BMD is predictive of future osteoporotic fracture.^33^^,^^37^^,^^38^ A third limitation of the study was that these analyses were limited to participants who attended a clinic visit. The clinic group were taller and the women were lighter than those who had only a home visit, and have also been found to have fewer health conditions.^23^ However there is no reason to suspect that the associations between pubertal markers and the bone outcomes should differ between the two groups at this stage of early ageing. A fourth limitation is that the sample were all born in the early post-war period; therefore our findings may not be generalizable to later-born cohorts. The decline in the age at menarche slowed from 2 months a decade to reach 12.8 years in the 1950s, and has been relatively stable since;^39^ however, we do see a secular decline in pubertal timing between NSHD participants born in 1946 and ALSPAC participants born 1991-92.^21^

## Conclusions

The association between the timing of puberty and BMD persists into early old age. The 9-10% lower peripheral trabecular vBMD in later compared with earlier maturers could be clinically important, given a rate of bone loss from midlife of 1-2% a year and the negative association between BMD and fracture.

## Funding

This work was supported by the UK Medical Research Council which provides core funding for the MRC National Survey of Health and Development and supports D.K., S.M. and R.H. by [MC_UU_12019/1, MC_UU_12019/4], K.W. by [U105960371] and T.J.C. by [MR/M012069/1]. The UK Medical Research Council and the Wellcome Trust (grant ref: 092731) and the University of Bristol provide core support for ALSPAC.

Key Messages

- Puberty is an important period for bone growth and mineral accrual, but evidence from previous studies on pubertal timing and adult bone health is inconclusive due to a lack of very long-term follow-up in studies with prospectively acquired pubertal indicators and gold-standard bone outcomes.
- This birth cohort study is unique in having prospective data acquired in adolescence on puberty, and bone outcomes in early old age derived from peripheral quantitative computed tomography and dual-energy X-ray absorptiometry, on 1359 men and women.
- Later puberty, based on reported menarche, on clinically assessed pubertal stage or on growth tempo based on growth curve analysis, was consistently associated with lower trabecular volumetric bone mineral density at the radius and lower areal bone mineral density of lumbar spine and total hip in participants aged 60-64 years.
- The 9-10% lower trabecular volumetric bone mineral density in later compared with earlier maturers may be clinically important, given midlife rates of bone loss and a negative association between bone mineral density and fracture.

## Supplementary Material

Supplementary Data

Supplementary Data
