# Supplementary material for: Pubertal timing and bone phenotype in early old age: findings from a British birth cohort study
Source: Int J Epidemiol. 2016 Jul 10;45(4):1113–24. doi: 10.1093/ije/dyw131 (PMC5075580; doi:10.1093/ije/dyw131)
Supplement: Supplementary Data [file dyw131_supplementary_data.zip › ije-2015-10-1387-File004.docx]

Supplementary Table 1. Mean and standard deviation (SD) for pQCT-derived and DXA-derived outcomes at 60-64 years by development of genitalia at 14.5 years. MEN

|  |  | Development of genitalia | | |  |  |
| --- | --- | --- | --- | --- | --- | --- |
|  |  | Advanced | Early | Pre-adolescent | Total  sample | p-value |
|  | N | Mean (SD) | Mean (SD) | Mean (SD) | Mean (SD) |  |
| pQCT |  |  |  |  |  |  |
| Distal CSA mm^2^ (4%) | 547 | 173 (34) | 169.6 (33) | 168 (37) | 171 (34) | .2 |
| Diaphysis CSA  Mm^2^ (50%) | 547 | 157 (23) | 152.5 (21) | 151 (29) | 154 (23) | .02 |
| Medullary CSA  Mm^2^ (50%) | 546 | 44 (14) | 41.2 (12) | 39 (16) | 42 (14) | .009 |
| Polar SSI  mm^3^ (50%) | 543 | 353 (71) | 342.5 (69) | 331 (69) | 348 (70) | .05 |
| Total vBMD  mg/cm^3^ (4%) | 547 | 392 (69) | 388.7 (66) | 403 (62) | 391 (67) | .9 |
| Trabecular vBMD mg/cm^3^ (4%) | 546 | 210 (42) | 200.5 (40) | 195 (43) | 205 (41) | .004 |
| Cortical vBMD  mg/cm^3^ (50%) | 547 | 1156 (36) | 1160.2 (33) | 1175 (22) | 1159 (35) | .02 |
| DXA |  |  |  |  |  |  |
| Lumbar spine aBMD  g/cm^2^ | 652 | 1.06 (0.18) | 1.04 (0.17) | 1.01 (0.17) | 1.05 (0.18) | .2 |
| Total hip aBMD  g/cm^2^ | 645 | 1.01 (0.15) | 0.985 (0.14) | 0.97 (0.17) | 1.00 (0.14) | .02 |
|  |  |  |  |  |  |  |

*p-values from regression models with logged bone outcomes and including genitalia development as a continuous variable

Supplementary Table 2. Mean and standard deviation (SD) for pQCT-derived and DXA-derived outcomes at 60-64 years by broken voice at 14.5 years. MEN

|  |  | Broken voice | | |  |  |
| --- | --- | --- | --- | --- | --- | --- |
|  |  | Completely broken | Starting to break | Not yet broken | Total  sample | p-value |
|  | N | Mean (SD) | Mean (SD) | Mean (SD) | Mean (SD) |  |
| pQCT |  |  |  |  |  |  |
| Distal CSA mm^2^ (4%) | 544 | 174 (35) | 171 (34) | 168 (31) | 171 (34) | .1 |
| Diaphysis CSA  Mm^2^ (50%) | 544 | 159 (23) | 152 (21) | 153 (23) | 155 (23) | .007 |
| Medullary CSA  Mm^2^ (50%) | 543 | 45 (14) | 41 (12) | 42 (14) | 43 (13) | .04 |
| Polar SSI  mm^3^ (50%) | 540 | 361 (75) | 340 (63) | 341 (70) | 348 (70) | .005 |
| Total vBMD  mg/cm^3^ (4%) | 544 | 396 (70) | 388 (67) | 391 (65) | 392 (67) | .5 |
| Trabecular vBMD mg/cm^3^ (4%) | 543 | 212 (43) | 205 (39) | 198 (41) | 206 (41) | .002 |
| Cortical vBMD  mg/cm^3^ (50%) | 544 | 1155 (37) | 1160 (33) | 1160 (33) | 1159 (35) | .2 |
| DXA |  |  |  |  |  |  |
| Lumbar spine aBMD  g/cm^2^ | 649 | 1.07 (0.18) | 1.05 (0.18) | 1.02 (0.16) | 1.05 (0.18) | .004 |
| Total hip aBMD  g/cm^2^ | 642 | 1.02 (0.14) | 0.99 (0.15) | 0.98 (0.14) | 1.00 (0.14) | .01 |
|  |  |  |  |  |  |  |

*p-values from regression models with logged bone outcomes and including voice broken as a continuous variable

Supplementary Table 3. Mean and standard deviation (SD) for pQCT-derived and DXA-derived outcomes at 60-64 years by development of pubic hair at 14.5 years. MEN

|  |  | Pubic hair | | |  |  |
| --- | --- | --- | --- | --- | --- | --- |
|  |  | Profuse | Sparse | None | Total  sample | p-value |
|  |  | Mean (SD) | Mean (SD) | Mean (SD) | Mean (SD) |  |
| pQCT |  |  |  |  |  |  |
| Distal CSA mm^2^ (4%) | 545 | 172 (34) | 171 (34) | 167 (34) | 171 (34) | .3 |
| Diaphysis CSA  Mm^2^ (50%) | 545 | 157 (23) | 153 (22) | 153 (21) | 155 (23) | .08 |
| Medullary CSA  Mm^2^ (50%) | 544 | 44 (15) | 41 (12) | 42 (13) | 43 (14) | .03 |
| Polar SSI  mm^3^ (50%) | 541 | 351 (72) | 345 (69) | 339 (67) | 347 (70) | .1 |
| Total vBMD  mg/cm^3^ (4%) | 545 | 397 (68) | 386 (65) | 385 (72) | 391 (67) | .08 |
| Trabecular vBMD mg/cm^3^ (4%) | 544 | 212 (41) | 201 (41) | 194 (39) | 205 (41) | <.001 |
| Cortical vBMD  mg/cm^3^ (50%) | 545 | 1155 (37) | 1163 (33) | 1159 (32) | 1159 (35) | .1 |
| DXA |  |  |  |  |  |  |
| Lumbar spine aBMD  g/cm^2^ | 650 | 1.06 (0.18) | 1.05 (0.18) | 1.01 (0.17) | 1.05 (0.18) | .04 |
| Total hip aBMD  g/cm^2^ | 643 | 1.00 (0.14) | 1.00 (0.15) | 0.96 (0.14) | 1.00 (0.14) | .05 |
|  |  |  |  |  |  |  |

*p-values from regression models with logged bone outcomes and including visible public hair as a continuous variable

Supplementary Table 4. Mean and standard deviation (SD) for pQCT-derived and DXA-derived outcomes at 60-64 years by development of axillary hair at 14.5 years. MEN

|  |  | Axillary hair? | |  |  |
| --- | --- | --- | --- | --- | --- |
|  |  | Yes | No | Total  sample | p-value |
|  | N | Mean (SD) | Mean (SD) | Mean (SD) |  |
| pQCT |  |  |  |  |  |
| Distal CSA mm^2^ (4%) | 544 | 172 (36) | 170 (32) | 171 (34) | .4 |
| Diaphysis CSA  Mm^2^ (50%) | 544 | 155 (22) | 152(23) | 155 (23) | .02 |
| Medullary CSA  Mm^2^ (50%) | 543 | 44 (14) | 42 (13) | 43 (14) | .1 |
| Polar SSI  mm^3^ (50%) | 540 | 354 (70) | 339 (70) | 347 (70) | .02 |
| Total vBMD  mg/cm^3^ (4%) | 544 | 397 (68) | 384 (65) | 391 (67) | .02 |
| Trabecular vBMD mg/cm^3^ (4%) | 543 | 212 (41) | 197 (41) | 205 (41) | <.001 |
| Cortical vBMD  mg/cm^3^ (50%) | 544 | 1157 (36) | 116 (33) | 1159 (35) | .1 |
| DXA |  |  |  |  |  |
| Lumbar spine aBMD  g/cm^2^ | 648 | 1.06 (0.18) | 1.03 (0.17) | 1.06 (0.18) | .006 |
| Total hip aBMD  g/cm^2^ | 641 | 1.01 (0.14) | 0.98 (0.15) | 1.00 (0.15) | .02 |
|  |  |  |  |  |  |

*p-values from regression models with logged bone outcomes and including axillary hair as a dichotomous variable

|  | Unadjusted | | | Adjusted for height and weight | | | + smoking and own SEP | | | + age at period cessation | | |
| --- | --- | --- | --- | --- | --- | --- | --- | --- | --- | --- | --- | --- |
|  | % diff | (95% CI) | P value | % diff | (95% CI) | P value | % diff | (95% CI) | P value | % diff | (95% CI) | P value |
| **Trabecular vBMD** (n=456) |  |  |  |  |  |  |  |  |  |  |  |  |
| Age at menarche | -10.2 | -19.7, -.70 | .035 | -6.2 | -15.8, 0.3 | .209 | -6.1 | -15.7, 0.4 | .216 | -6.0 | -15.7, 0.4 | .227 |
| **Total hip aBMD**  (n=566) |  |  |  |  |  |  |  |  |  |  |  |  |
| Age at menarche | -9.2 | -14.1, -4.1 | <.001 | -4.8 | -9.2, -0.4 | .033 | -4.6 | -9.1, -0.3 | .037 | -4.8 | -9.2, -0.4 | .033 |
| **Lumbar spine aBMD**  **(**n=566) |  |  |  |  |  |  |  |  |  |  |  |  |
| Age at menarche | -8.9 | -14.6, -3.1 | .003 | -6.1 | -11.7, -0.5 | .032 | -6.0 | -11.6, -0.4 | .035 | -6.3 | -11.9, -0.7 | .028 |

Supplementary Table 5. Percentage difference in trabecular vBMD, total hip and lumbar spine aBMD by age at menarche in women with known age at period cessation, unadjusted and adjusting for body size, then smoking and adult SEP, and then age at period cessation.
