# Supplementary material for: Pubertal timing and bone phenotype in early old age: findings from a British birth cohort study
Source: Int J Epidemiol. 2016 Jul 10;45(4):1113–24. doi: 10.1093/ije/dyw131 (PMC5075580; doi:10.1093/ije/dyw131)
Supplement: Supplementary Data [file supp_45_4_1113__index.html]

Pubertal timing and bone phenotype in early old age: findings from a British birth cohort study — Supplementary Data 

# Pubertal timing and bone phenotype in early old age: findings from a British birth cohort study

## Supplementary Data

files

- Supplementary Data - docx file
